# Gender gap-related issues among mothers revealed by a comparative study of adolescent hikikomori between Japan and France

**DOI:** 10.1192/j.eurpsy.2023.631

**Published:** 2023-07-19

**Authors:** Y. Hamasaki, G. Dorard, N. Tajan, N. Pionnié‑Dax

**Affiliations:** ^1^Kyoto Women’s University, Kyoto, Japan; ^2^Université de Paris, Paris, France; ^3^Kyoto University, Kyoto, Japan; ^4^EPS ERASME, Antony, France

## Abstract

**Introduction:**

Around 2010, the number of hikikomori cases increased rapidly. Hikikomori is a global problem that characterizes the current era, and has become an increasingly deep-rooted social issue that affects the younger generation, especially during the coronavirus disease-2019 (COVID-19) pandemic. In our previous comparative study of adolescent hikikomori between Japan and France (Hamasaki et al. BMC Psychiatry 2022; 22, 477), we investigated its psychopathology, including potential cultural influencing factors. The study showed no difference in terms of psycho-behavioral characteristics of hikikomori between Japan and France. However, the sociocultural factors that make hikikomori more severe differed between the two countries, i.e., in Japan: lack of communication between parents, in France: lack of communication between the family and the community.

**Objectives:**

Since these differences in sociocultural factors are closely related to the social context in which the mothers were placed, the factors in terms of maternal gender issues were examined, along with reviewing previous studies.

**Methods:**

Statistical data from the “Global Gender Gap Report 2022” of the World Economic Forum, the “Towards real gender equality 2021” of the Ministry of Gender Equality, Diversity, and Equal Opportunities of France, the “Women and Men in Japan 2020” of the Gender Equality Bureau, Cabinet Office, Government of Japan, and other sources were evaluated. Further, previous literature on the family environmental factors of hikikomori were reviewed.

**Results:**

An absent father, a subsequent mother-child closeness and over-interference, and the inhibition of children’s independence, have been repeatedly mentioned in studies as factors leading to hikikomori. The time spent on housework and childcare by Japanese men is at the lowest level globally (Japan’s gender gap index ranks 116^th^ out of 146 countries, the lowest among the seven major G7 nations). In Japan, where generally little cooperation exists between parents, particularly in those families where communication between parents is self-rated as relatively poor, the above factors may surpass the threshold for triggering hikikomori. In France (ranked 15^th^ in gender gap index), the isolation of mothers and children from society is an important factor associated with hikikomori. Adequate social participation of the mother may be a protective factor against hikikomori.

**Conclusions:**

Gender gap-related issues among mothers may be involved in the root of the hikikomori problem. Hikikomori has emerged from various socio-familial factors. Further studies are warranted to determine the causal relationships of these factors with the onset and severity of hikikomori.

**Disclosure of Interest:**

None Declared

